# Investigation of the Interaction between Patulin and Human Serum Albumin by a Spectroscopic Method, Atomic Force Microscopy, and Molecular Modeling

**DOI:** 10.1155/2014/734850

**Published:** 2014-07-08

**Authors:** Li Yuqin, You Guirong, Yang Zhen, Liu Caihong, Jia Baoxiu, Chen Jiao, Guo Yurong

**Affiliations:** ^1^College of Pharmacy, Taishan Medical University, Taian 271016, China; ^2^College of Clinic, Taishan Medical University, Taian 271016, China; ^3^College of Life Science and Technology, China Pharmaceutical University, Nanjing 210009, China; ^4^College of Food Engineering and Nutritional Science, Shaanxi Normal University, Xi'an 710062, China

## Abstract

The interaction of patulin with human serum albumin (HSA) was studied in vitro under normal physiological conditions. The study was performed using fluorescence, ultraviolet-visible spectroscopy (UV-Vis), circular dichroism (CD), atomic force microscopy (AFM), and molecular modeling techniques. The quenching mechanism was investigated using the association constants, the number of binding sites, and basic thermodynamic parameters. A dynamic quenching mechanism occurred between HSA and patulin, and the binding constants (*K*) were 2.60 × 10^4^, 4.59 × 10^4^, and 7.01 × 10^4^ M^−1^ at 288, 300, and 310 K, respectively. Based on fluorescence resonance energy transfer, the distance between the HSA and patulin was determined to be 2.847 nm. The Δ*G* ^0^, Δ*H* ^0^, and Δ*S* ^0^ values across various temperatures indicated that hydrophobic interaction was the predominant binding force. The UV-Vis and CD results confirmed that the secondary structure of HSA was altered in the presence of patulin. The AFM results revealed that the individual HSA molecule dimensions were larger after interaction with patulin. In addition, molecular modeling showed that the patulin-HSA complex was stabilized by hydrophobic and hydrogen bond forces. The study results suggested that a weak intermolecular interaction occurred between patulin and HSA. Overall, the results are potentially useful for elucidating the toxigenicity of patulin when it is combined with the biomolecular function effect, transmembrane transport, toxicological, testing and other experiments.

## 1. Introduction

Patulin is a toxic secondary metabolite produced by a variety of food spoilage fungi, particularly by* Penicillium*, Aspergillus genera, and* Byssochlamys* species [1]. Different foods, including fruits and grains, especially apple and its juice, can be affected by these fungi and become contaminated with patulin [2]. The highest patulin production has been observed at storage temperatures between 4 and 25°C [3–5]. Patulin has a strong affinity for sulfhydryl groups, inhibiting the activity of many enzymes [6, 7]. It also has strong antibiotic properties [8] and was used to treat the common cold in the 1940s. Acute toxicity after high dosing in animals is expressed as agitation, convulsions, dyspnea, pulmonary congestion, edema, ulceration, hyperemia, and gastrointestinal tract distension [9–11]. Nausea, vomiting, gastrointestinal disturbance, and kidney damage have been reported in humans [12, 13]. Patulin has been reported to be a genotoxic, reprotoxic, embryotoxic, and immunosuppressive compound [14–18]. To establish guidelines for human exposure to patulin, the JECFA lowered the provisional maximum tolerable daily intake (PMTDI) of patulin from 1 to 0.4 mg/kg body mass/day based on a no-observed-effect level (NOEL) of 43 mg/kg body mass/day and the use of a 100-fold safety factor [6].

Human serum albumin (HSA) containing 585 amino acid residues is the most abundant protein constituent of the circulatory system, contributing significantly to physiological functions as carrier proteins [19]. HSA contains three homologous *α*-helical domains (I-III). Each of these domains is divided into A and B subdomains that contain six and four *α*-helices, respectively [20, 21]. Based solely on this sole structure, HSA has an ability to bind an unusually broad spectrum of ligands such as inorganic ions, various drugs, amino acids, and fatty acids [22]. Binding to HSA facilitates the transport of these ligands throughout the circulation [23]. Moreover, these bindings appear to affect the secondary and tertiary structure of albumin [24]. Without a doubt, the interaction of any toxicant with HSA affects the transport of nutrients and drugs. Recently, studies have been conducted on the binding of organic contaminants or toxins to HSA, for example, Arazine, ochratoxin A, methyl parathion, arsenic, perfluorooctanoic acid and phthalate esters, deoxynivalenol, and 2-mercaptobenzimidazole [25–32].

In this study, the interaction of patulin with HSA was investigated using steady-state and time-resolved fluorescence spectroscopy, UV-Vis spectroscopy, circular dichroism (CD) spectroscopy, atomic force microscopy (AFM), and molecular modeling.

## 2. Materials and Methods

### 2.1. Materials

**Figure 1:**
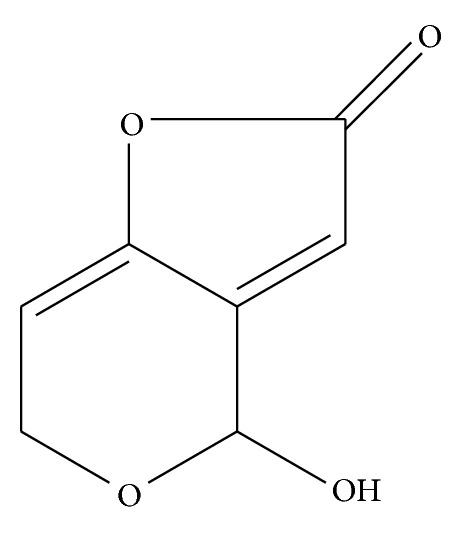
The chemical structure of patulin.

HSA (fatty acid free < 0.05%) was purchased from Sigma Chemical Company (USA), and patulin (Figure 1, analytical grade) was purchased from J & K Technology Co., Ltd (USA). A 0.05 mol*·*L^−1^ Tris-HCl (pH 7.4) buffer solution was used to maintain the solution pH. A 15 *μ*mol*·*L^−1^ HSA stock solution was prepared in Tris-HCl buffer solution and stored in the dark at 4°C. Dilutions of the HSA stock with Tris-HCl buffer solution were prepared immediately before use. A 1.0 mol*·*L^−1^ NaCl solution was used to maintain an ionic strength of 0.1 M. A 1.0 mmol*·*L^−1^ patulin solution was prepared in double-distilled water. All other reagents were of analytical grade, and double-distilled water was used throughout all experiments.

### 2.2. Apparatuses and Methods

#### 2.2.1. Steady-State and Synchronous Fluorescence Spectroscopy

Steady-state fluorescence spectra were obtained using an F-4500 fluorescence spectrometer (Hitachi, Japan). The excitation and emission slit widths were 5 nm. An excitation wavelength of 295 nm and emission wavelengths between 300 and 500 nm were recorded.

The interactions between patulin and HSA were measured by the fluorescence method. The appropriate concentrations of HSA, NaCl, and patulin—15 *μ*mol*·*L^−1^, 1.0 mol*·*L^−1^, and 1.0 mmol*·*L^−1^, respectively—were added to a 10 mL volumetric flask; next, the mixture was diluted to graduation with Tris-HCl buffer solution. All experiments were performed at 288, 300, and 310 K. The method of synchronous fluorescence spectroscopy was carried out as described above.

#### 2.2.2. Time-Resolved Fluorescence Spectroscopy

Time-resolved fluorescence spectra were measured by steady-state spectroscopy (FLS-920) (Edinburgh Instruments) using a standard time-correlated and single-photon counting scheme. Samples were excited with a subnanosecond pulsed diode laser at a repetition rate of 10 MHz at 295 nm. Fluorescence spectra were detected three times at 345 nm and at 288 K. The decay of the fluorescence intensity was determined with the instrument-specific software based on tail fitting. The multiexponential values were assessed by considering the reduced chi-square value (*χ* ^2^ < 1.10) and the random distribution of the weighted residuals near zero.

#### 2.2.3. UV-Vis Absorption Spectra

Absorption spectra were obtained with a UV-2450 UV-Vis spectrometer (Shimadzu, Japan) at 288 K in the range of 200–500 nm using a 1 cm quartz cell. The method employed was the same as that described in Section 2.2.1.

#### 2.2.4. CD Spectra

CD measurements were collected with a JASCO J-810 circular dichroism spectrometer (Japan) at 288 K using a 0.1 cm quartz cell. The HSA concentration was 1.5 *μ*mol*·*L^−1^, and the scanning speed was 200 nm min^−1^. The buffer solution was used as a blank and was automatically subtracted from the samples after scanning. Each sample was measured three times at a bandwidth of 1.0 nm. The CD results were expressed in units of millidegrees.

#### 2.2.5. Atomic Force Microscopy

AFM was carried out with a MultiMode Nanoscope IIIa (USA) equipped with a normal NP probe. The spring constant of the cantilever was 0.32 N/m, and the typical imaging resonance frequency of the fluid was 7–9 kHz. All of the samples were imaged in fluid contact mode with an O-ring liquid cell. Samples were prepared as follows: (1) free HSA with 30 *μ*L of 1.5 *μ*mol*·*L^−1^ HSA was added to a mica substrate and incubated for 10 min at 288 K before washing with water; (2) HSA-patulin complexes with free HSA samples were prepared as described in step (1) prior to adding 20 *μ*L of a 15 *μ*mol*·*L^−1^ patulin solution, incubating for 15 min, washing with water, drying under N_2_ for 4 min, and imaging in air.

#### 2.2.6. Molecular Docking

The 3D structure of the ligand was constructed with standard bond lengths and bond angles using the molecular modeling software program SYBYL8.0 (Tripos Inc., St. Louis, USA) for Linux. Geometry optimization was performed using the standard Tripos forcefield [33] with a distance-dependent dielectric function and an energy gradient of 0.001 kcal/mol. Gasteiger-Hückel charges [34] were used.

Molecular docking was implemented using MOE2009 for Windows (Chemical Computing Group Inc., Montreal, Canada). The available X-ray structure of HSA complexed with R-warfarin (PDB code: 1H9Z) was applied in this work as the receptor. Hydrogen atoms were added to the PDB file. Then, the 1H9Z complex was handled in LigX (a module of the MOE software) to meet the docking requirements. The conformer with the lowest *S* value was used for further analysis.

## 3. Results and Discussion

### 3.1. Analysis of Fluorescence Quenching of HSA by Patulin

**Figure 2:**
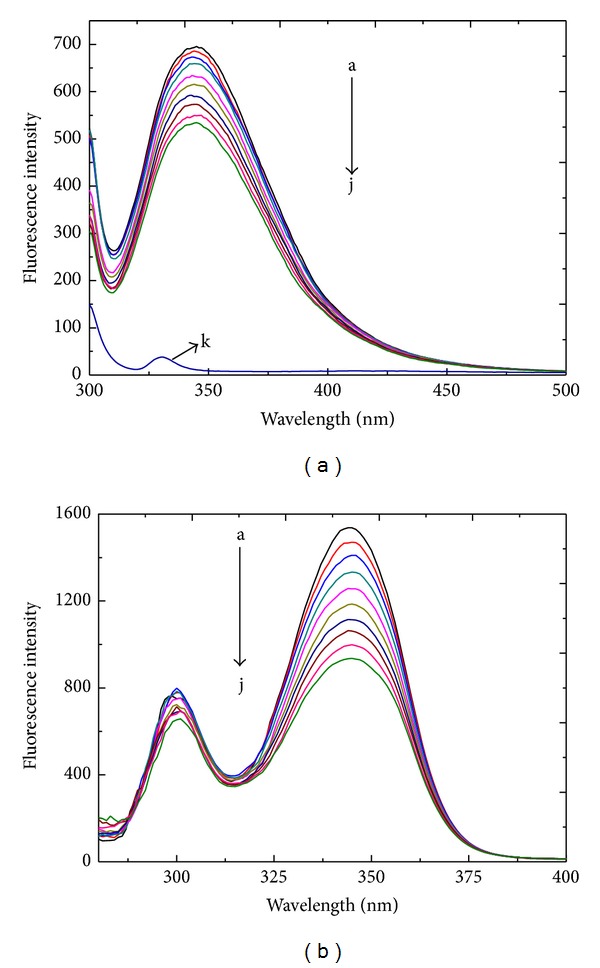
The fluorescence emission (a) and synchronous (b) spectra of the patulin-HSA system. The concentration of HSA was 1.5 *μ*M, whereas the patulin concentrations were 0, 3.33, 6.67, 10.0, 13.3, 16.7, 20, 23.3, 26.7, and 30.0 *μ*M from a to j, respectively. (k) [HAS] = 0 *μ*M; [patulin] = 10.0 *μ*M. Trisbuffer, pH = 7.4, *T* = 288 K, and *λ*_ex_ = 295 nm.

The intrinsic fluorescence of HSA is derived mainly from tryptophan (Trp), tyrosine (Tyr), and phenylalanine (Phe) residues. Phe residue fluorescence has a very low quantum yield, and Tyr residue fluorescence is nearly quenched when the residue is ionized or near an amino or carboxyl group of Trp. The only tryptophan residue (Trp-214) in HSA is located in domain II, Site I. The fluorescence of Trp-214 is sensitive to the ligand to which the residue binds. Therefore, Trp-214 is often used as a probe to investigate the interaction of small ligands with HSA.  Figure 2(a) shows the fluorescence spectra of HSA with different patulin concentrations at *λ*_ex_ 295 nm. The maximum emission wavelength (*λ*_em_) of free HSA was 345 nm. Its intensity gradually decreased with the concentration of patulin, and the peak position was slightly blue shifted. This result indicates that patulin could quench the intrinsic fluorescence of HSA and that Trp-214 was more polar and hydrophobic after the addition of patulin.

Synchronous fluorescence is a very useful tool for investigating the microenvironments of fluorophore functional groups. According to Miller [35], the fluorescence of HSA with a Δ*λ* (Δ*λ* = *λ*_em_ − *λ*_ex_) of 60 nm is characteristic of the alteration of the polarity of the microenvironment surrounding Trp-214 residues. The effect of patulin on synchronous fluorescence spectra is illustrated in Figure 2(b). The fluorescence intensity of Trp residues decreased with an increase in patulin concentration. The results correspond to those shown in the fluorescence spectra of Figure 2(a), suggesting that the microenvironment of Trp-214 was made more hydrophobic.

To further confirm the quenching mechanism, the fluorescence quenching data were analyzed with the Stern-Volmer equation:

(1) F0F=1+KSV[Q],

where *F*_0_ and *F* are the fluorescence intensity in the absence and presence of a quencher, respectively, and [*Q*] is the quencher concentration. *K*_SV_ is the Stern-Volmer quenching constant. The *K*_SV_ values at different temperatures are listed in Table 1. According to the fluorescence quenching mechanisms, a higher temperature would generally result in faster diffusion and the dissociation of weakly bound complexes [36]. The *K*_SV_ values increased with increasing temperature, which suggests that a dynamic quenching mechanism occurred between HSA and patulin.

**Figure 3:**
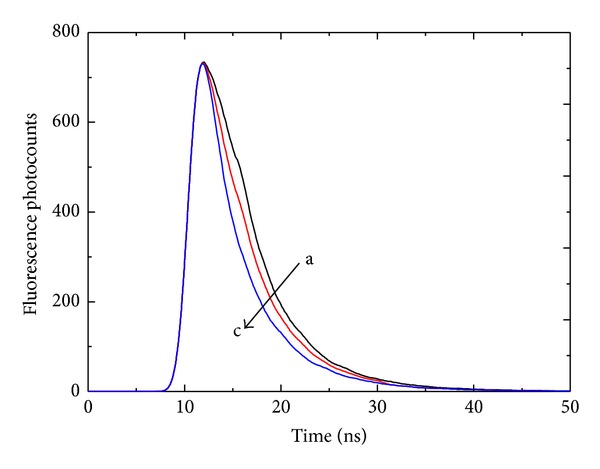
The time-resolved fluorescence decay of the patulin-HSA system. *C*_HSA_ = 1.5 *μ*M. Patulin concentrations were 0.0, 1.5, and 3.0 *μ*M from a to c, respectively. Trisbuffer, pH = 7.4, *T* = 288 K, and *λ*_ex_ = 295 nm.

Time-resolved fluorescence spectroscopy is a tool that can be used to investigate the interaction between ligands and proteins. The fluorescence lifetime can be used to directly differentiate between dynamic and static quenching. The lifetime of static quenching does not depend on the quencher concentration (i.e., 〈*τ*_0_〉 = 〈*τ*〉) [37, 38]. The time-resolved fluorescence spectra of the free HSA and patulin-HSA complexes are shown in Figure 3, and the fluorescence decay parameters are listed in Table 2. The fluorescence decay of the unquenched Trp-214 was biexponential (*χ* ^2^ = 1.007) (with *τ*_1_ = 3.06 ns [*α*_1_ (fraction) = 0.64], *τ*_2_ = 7.80 ns [*α*_2_ (fraction) = 0.36], and 〈*τ*〉 = 5.37 ns). These results are in agreement with those obtained for Trp-214 by Helms et al. [39]. As indicated in Table 2, the average fluorescence lifetime of Trp-214 decreased slightly with increasing patulin concentration. Therefore, these results suggest that the HSA-patulin quenching mechanism was dynamic.

### 3.2. The Binding Constant and Binding Site

The apparent binding constant (*K*_*A*_) and the binding site number (*n*) for the patulin-HSA complex were calculated by the following equation:

(2) lgF0−FF=lgKA+nlg[Q],

where, *F*_0_ and *F* indicate the fluorescence intensity of the system in the absence and presence of a quencher and [*Q*] is the total quencher concentration. The values of *K*_*A*_ and *n* for patulin-HSA are also listed in Table 1. The *K*_*A*_ values increased with temperature, which indicates that the patulin-HSA quenching mechanism was dynamic. This result agrees with the results described in Section 3.1.

### 3.3. Binding Mode

There are several types of noncovalent interaction modes between proteins and ligands, such as hydrogen bonds, van der Waals forces, hydrophobic interaction forces, and electrostatic forces [40]. The thermodynamic parameters, enthalpy change (Δ*H* ^0^), and entropy change (Δ*S* ^0^) of reaction are important for confirming what binding mode is active. Therefore, the temperature dependence of the binding constant was studied. Δ*H* ^0^ and Δ*S* ^0^ were obtained using the van't Hoff equations [(3) and (4)]:

(3) ln⁡K=−ΔH0RT+ΔS0R,

where *K* is the binding constant obtained from (1) and *R* is the universal gas constant. Δ*H* ^0^ and Δ*S* ^0^ were obtained from (4). Then, the free energy change (Δ*G* ^0^) was estimated using

(4) ΔG0=ΔH0−TΔS0.

The Δ*H* ^0^, Δ*S* ^0^, and Δ*G* ^0^ values are also listed in Table 1. The Δ*H* ^0^ and Δ*S* ^0^ values of patulin-HSA were 8.06 kJ mol^−1^ and 106.0 J mol^−1^ K^−1^, respectively. According to the results of Timasheff and Subramanian regarding various protein interactions [41, 42], a positive Δ*S* ^0^ value is frequently regarded as evidence of a hydrophobic ligand-protein interaction considering the theory of the structure of water molecules. Thus, the hydrophobic interaction played a major role in the binding of patulin and HSA.

### 3.4. Energy Transfer between HSA and Patulin

**Figure 4:**
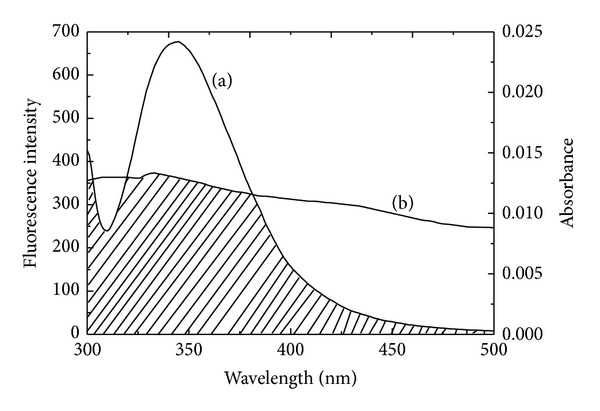
The overlap of (a) the absorption spectra of patulin and (b) the fluorescence emission spectrum of HSA. *C*_HSA  _ = 1.5 *μ*M and *C*_patulin_ = 1.5 *μ*M (288 K, pH = 7.4, and *λ*_ex_ = 295 nm).

The molecular distance *r* between HSA and patulin can be determined using the Förster energy transfer theory. The overlap of patulin's UV absorption spectrum with its fluorescence spectrum is shown in Figure 4. Based on the Förster energy transfer theory, the distance *r* can be calculated using

(5) E=1−FF0=R06(R06+r6),

where *E* is the efficiency of the transfer between HSA and patulin. *F* and *F*_0_ are the fluorescence intensities of HSA before and after the addition of patulin, respectively. *r* is the distance between patulin and HSA, and *R*_0_ is the critical distance at which the transfer efficiency is 50% [30]. The value of *R*_0_ is calculated using

(6) $$\begin{array}{c} R_{0}^{6}=8.8\times 10^{-25}K^{2}n^{-4}\phi J, \end{array}$$

where *K* ^2^ is the spatial orientation factor of the dipole, *n* is the refractive index of the medium, and *ϕ* is the fluorescence quantum yield of HSA in the absence of an acceptor. In addition, *J* is the overlap integral of the HSA and patulin fluorescence spectra. The value of *J* is calculated using

(7) J=∑F(λ)ε(λ)λ4Δλ∑F(λ)Δλ,

where *F*(*λ*) denotes the fluorescence intensity of either donor at wavelength *λ*, and *ε*(*λ*) denotesthe molar absorption coefficient of patulin at wavelength *λ* (cm mol^−1^). In this study, the value of *J* was 1.896 × 10^−15^ *·* cm^3^ *·*L*·*mol^−1^ and the value of *E* was 0.0803. For HSA, the *K* ^2^ is equal to 2/3 and the *ϕ* is equal to 0.118 for an *n* value of 1.336. Thus, *R*_0_ was 1.896 nm, and *r* was equal to 2.847 nm. Clearly, the distance between HSA and patulin was less than 8 nm and within 0.5*R*_0_ < *r* < 1.5*R*_0_. This result is in accordance with the Förster energy transfer theory and indicates that energy transfer occurred between patulin and HSA.

### 3.5. Effect of Patulin on the Secondary Structure of HSA

#### 3.5.1. UV-Vis Results

**Figure 5:**
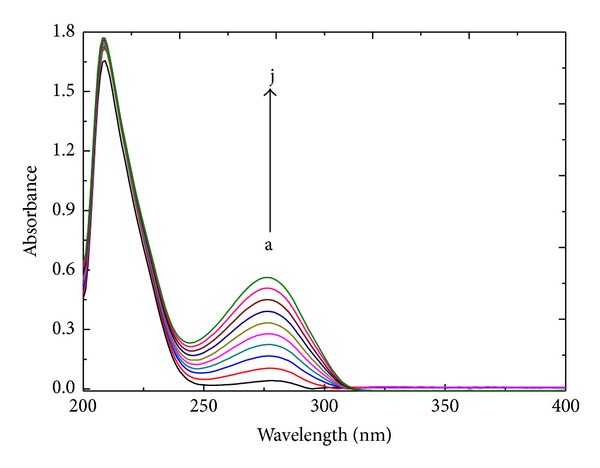
UV absorption spectra of the patulin-HSA system. The concentration of HSA was 1.5 *μ*M, whereas the patulin concentrations were 0, 3.33, 6.67, 10.0, 13.3, 16.7, 20, 23.3, 26.7, and 30.0 *μ*M from a to j, respectively. Trisbuffer, pH = 7.4, and *T* = 288 K.

Absorption at approximately 210 nm is indicative of the a-helix structure of HSA.  Figure 5 shows the UV absorption spectra of HSA in the absence and presence of patulin.  Figure 5 shows that HSA exhibited strong absorbance at 209 nm and weak absorbance at 280 nm. The intensities of these two absorbance peaks increased with the addition of patulin, and there was little shift wavelengths. These two results clearly indicate that there was an interaction between patulin and HSA.

#### 3.5.2. CD Results

**Figure 6:**
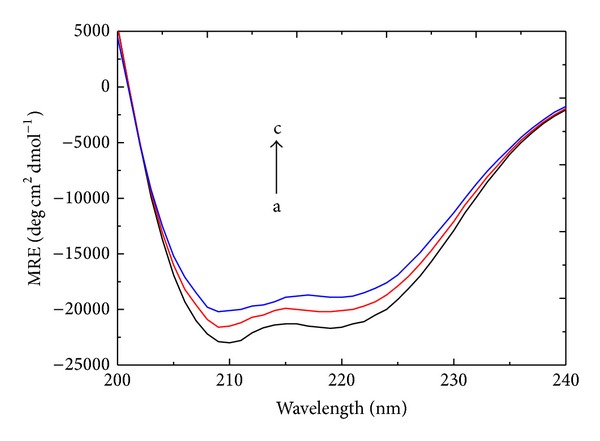
The CD spectra of the patulin-HSA system. Patulin concentrations were 0.0, 1.5, and 3.0 *μ*M from a to c, respectively. *C*_HSA_ = 1.5 *μ*M, pH = 7.4, and *T* = 288 K.

CD spectroscopy is a quantitative technique that is used to study the conformation of proteins in aqueous solutions. The CD spectra of HSA exhibited two negative bands at 208 and 219 nm, which characterize the *α*-helical structure of the protein [23]. The CD spectra of the free HSA and HSA-patulin complexes are shown in Figure 6. The secondary structure of HSA was calculated using SELCON3 (http://dichroweb.cryst.bbk.ac.uk/html); the results are listed in Table 3.

Table 3 shows that the free HSA had a high *α*-helix content of 55.3% and contained 8.7% *β*-sheets, 15.3% *β*-turns, and 20.7% random coils. With patulin addition, the band intensity increased at all wavelengths and no significant peak shifts were observed; furthermore, the *α*-helix content of HSA decreased to 50.7%, accompanied by an increase in the contents of *β*-sheets and *β*-turns to 10.6% and 17.4%. The content of random coils was only slightly altered when the molar concentration ratio of patulin to HSA was increased from 0 : 1 to 2 : 1. Thus, the binding of HSA with patulin caused conformational changes in HSA and the loss of *α*-helix stability.

#### 3.5.3. The Interactions between Patulin and HSA Based on Atomic Force Microscopy

**Figure 7:**
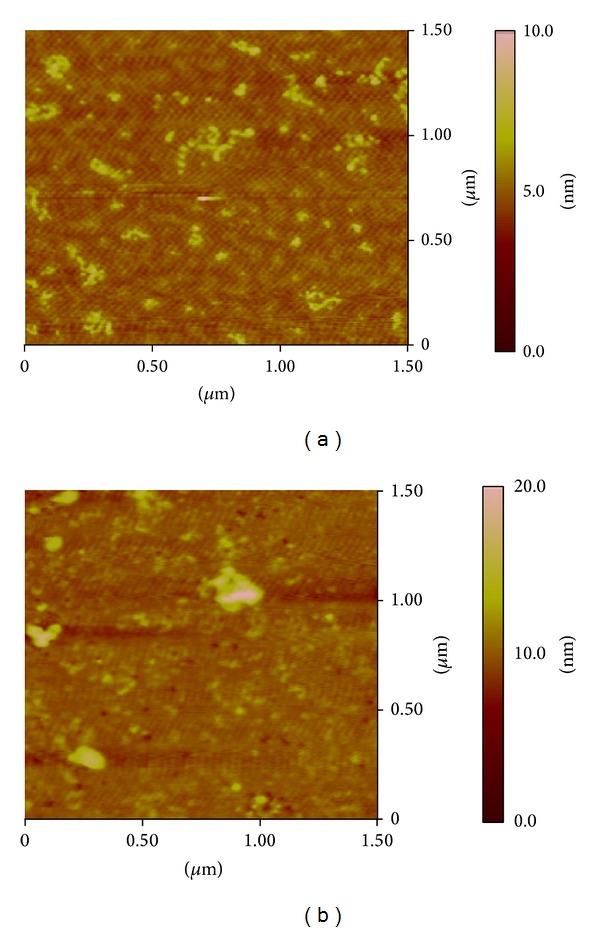
(a) An AFM topography image of free HSA and (b) an AFM topography image of the HSA-patulin complex. Samples were adsorbed onto mica under tapping mode in a Tris-HCl buffer solution, and the scan size of the image is 1.5 *μ*m × 1.5 *μ*m.

To study the changes in HSA topography with the addition of patulin, the free HSA and HAS-patulin complexes were imaged by AFM in triplicate.  Figure 7 shows the AFM results obtained for the free HSA and its complexes.

As shown in Figure 7(a), HSA was adsorbed evenly on the mica surfaces. The mean height of the individual HSA molecules was 5.00 ± 2.47 nm. These dimensions are consistent with the results of previous AFM studies [43]. After the addition of patulin, the HSA molecule became swollen, and the mean height of HSA reached 15.2 ± 3.05 nm (Figure 7(b)). The aggregation of the HSA molecules is also shown in Figure 7(b). The microenvironment around the HSA became more hydrophobic after interacting with patulin. To minimize any factors adversely affecting the formation of a stable structure, the HSA molecule reduced its surface area of contact with water by molecular aggregation. These results reveal that a hydrophobic interaction between HSA and patulin may occur.

### 3.6. Molecular Modeling

**Figure 8:**
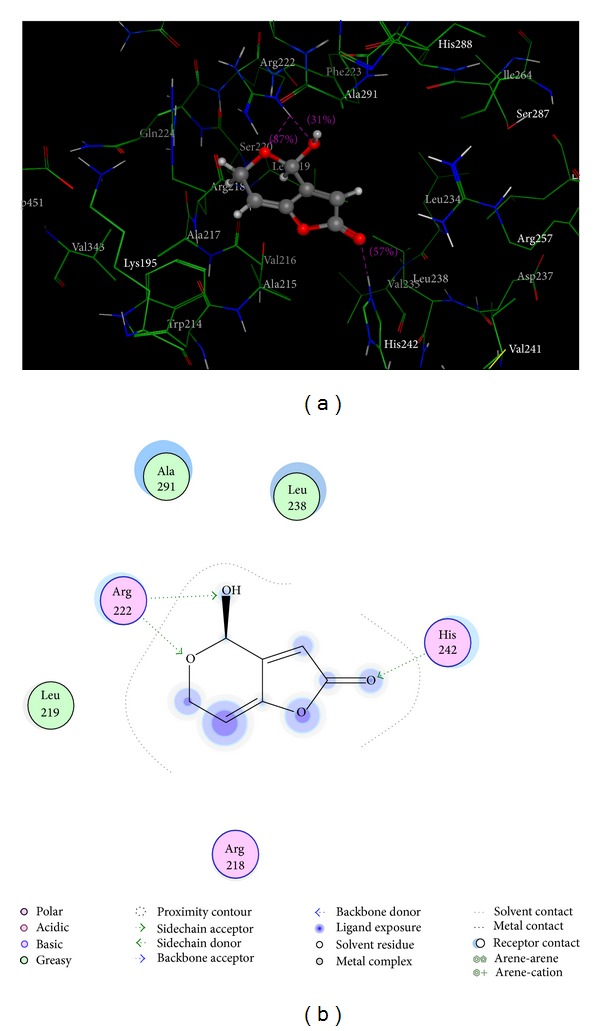
(a) The interaction mode between patulin and HAS. (b) A projection of 8a. The HSA residues are represented by lines, and the patulin structure is represented by a ball-and-stick model. Hydrogen bonds between patulin and HSA are represented by dashed lines.

Crystal structure analyses indicated that HSA contains the three following domains, which are structurally similar (I–III): I (residues 1–195), II (196–383), and III (384–585). The principal ligand binding sites on HSA are located in the hydrophobic cavities in subdomains IIA and IIIA, which correspond to sites I and II (according to the terminology of Sudlow et al.) [44]. Site I of HSA, which is a preferred binding pocket within the IIA subdomain [44, 45], shows affinity for warfarin, phenylbutazone, and other drugs. In addition, site II shows affinity for ibuprofen, flufenamic acid, and other ligands. To obtain more information about the patulin-HSA binding sites, molecular docking was used to simulate the patulin-HSA interaction.  Figure 8 shows the patulin-HSA interaction mode.  Figure 8(b) is a projection of Figure 8(a).

As shown in Figure 8, the patulin molecule was located within the binding pocket of site I (IIA), and the two rings were coplanar. The patulin molecule was inserted in the hydrophobic cavity that was formed by Ala291, Leu238, and Leu219. In addition, the pyran ring oxygen and 4-hydroxyl formed two hydrogen bonds with Arg222, and a furan ring carbonyl oxygen formed a hydrogen bond with His242. The docking results indicate that the interaction between patulin and HSA was dominated by hydrophobic forces accompanying with hydrogen bonds. In addition, several polar residues were observed near the patulin molecule, which played a subordinate role in stabilizing the ligand molecule through electrostatic interactions. For patulin-HSA, the calculated binding energy Δ*G* ^0^ was −26.68 kJ mol^−1^, which is near the experimental value [−22.46 kJ mol^−1^ at 288 K], which further conformed the experimental results and demonstrated that the spontaneous binding between patulin and HSA occurred.

## 4. Conclusions

In this study, the interactions of patulin with HSA were investigated under simulated physiological conditions using different spectroscopic methods: AFM and molecular modeling. Steady-state and time-resolved fluorescence spectra suggest that a dynamic quenching mechanism occurred in patulin-HSA complexes. The binding parameters for the reaction were determined using the Stern-Volmer equation. The thermodynamic parameters obtained at different temperatures and the molecular modeling results indicate that hydrophobic interaction was the dominant binding force and also suggest the formation of hydrogen bonds between patulin and HSA. This finding is expected to elucidate the toxigenicity of patulin when it is combined with the biomolecular function effect, transmembrane transport, toxicological testing, and other experiments.

## Figures and Tables

**Table 1:** Binding and thermodynamic parameters for the patulin-HSA interaction at different temperatures in Trisbuffer (pH = 7.4).

*T*/K	Equation (2)		Equation (3)		*n *	Δ*H* ^0^/kJmol^−1^	Δ*S* ^0^/J mol^−1^	Δ*G* ^0^/kJ mol^−1^
	*K*_SV_/M^−1^	*R *	*K*_*A*_/M^−1^	*R *				
288	1.17 × 10^4^	0.9961	2.60 × 10^4^	0.9769	1.10			−22.46
300	1.40 × 10^4^	0.9985	4.59 × 10^4^	0.9914	1.17	8.06	106.0	−23.73
310	1.48 × 10^4^	0.9972	7.01 × 10^4^	0.9982	1.34			−24.79

**Table 2:** Fluorescence decay fitting parameters for the patulin-HSA system in Trisbuffer (pH = 7.4).

Substance	*τ*_1_ (ns)	*α*_1_	*τ*_2_ (ns)	*α*_2_	*χ* ^2^	*τ* (ns)
HSA	3.06	0.64	7.80	0.36	1.007	5.37
Patulin-HAS (1 : 1)	2.82	0.51	7.26	0.49	1.020	5.28
Patulin-HAS (2 : 1)	2.55	0.47	6.19	0.53	1.024	5.11

**Table 3:** Secondary structure determination for free HSA and its drug complexes in Trisbuffer (pH = 7.4) at different molar concentration ratios for HSA and patulin.

Molar ratio (patulin-HSA)	*α*-helical (%)	*β*-sheet (%)	*β*-turn (%)	Random coil (%)
0 : 1	55.3	8.7	15.3	20.7
1 : 1	52.7	9.1	15.6	22.6
2 : 1	50.7	10.6	17.4	21.3

## Abbreviations

PMTDI: The provisional maximum tolerable daily intake
NOEL: A no-observed-effect level
HSA: Human serum albumin
CD: Circular dichroism
AFM: Atomic force microscopy
Trp: Tryptophan
Tyr: Tyrosine
Phe: Phenylalanine.
